# Physicochemical, Microbiological, and Toxicological Characterization of Pâté Prepared from the Meat and Liver of Bullfrog (*Aquarana catesbeiana*) Carcasses

**DOI:** 10.3390/foods12224064

**Published:** 2023-11-08

**Authors:** Luiz Rogério G. Magalhães, Victor F. Moebus, Airton A. Castagna, Marcos Aronovich, Carlos Eduardo R. Coutinho, Saida Favotto, Edi Piasentier, Luiz A. M. Keller, Eliana F. M. Mesquita

**Affiliations:** 1 Post Graduation Program in Hygiene Veterinary and Technology Processes of Animal Product Origins, Federal Fluminense University, Niterói 24230-321, Brazil; magalhaesluizrogerio@gmail.com; 2 Independent Researcher, Niterói 24120-191, Brazil; airtoncastagna@gmail.com; 3 Veterinary School, Castelo Branco University, Campus Penha, Rio de Janeiro 21012-351, Brazil; marcosaronovich@gmail.com; 4 Fisheries Foundation Institute of the State of Rio de Janeiro (FIPERJ), Niterói 24030-020, Brazil; carlos.coutinho@id.uff.br; 5 Department of Agricultural, Food, Environmental and Animal Sciences, University of Udine, 33100 Udine, Italy; saida.favotto@uniud.it (S.F.); edi.piasentier@uniud.it (E.P.); 6 Zootechnics and Sustainable Agro-Socio-Environmental Development Department, Federal Fluminense University, Niterói 24020-141, Brazil; luiz_keller@id.uff.br; 7 Food Technology Department, Federal Fluminense University, Niterói 24230-321, Brazil; elianamesquista@id.uff.br

**Keywords:** liver pâté, seafood technology, bullfrog torso, minced meat, spoilage products

## Abstract

The development of balanced, healthy, ready-to-consume, and easy-to-prepare products has led to the development of new food technologies. Despite their high commercial value, bullfrog (*Aquarana catesbeiana*) carcasses result in low yields, with the thighs being the most marketed in comparison to other carcass portions. In this sense, liver pâté is a traditional food consumed worldwide, mainly in European countries, and may be prepared by incorporating bullfrog meat by-products and certain viscera. In this context, the aim of the present study was to develop a pâté product based on a mixture comprising 50% grounded bullfrog torso meat and 50% liver paste, with each treatment incorporating 10% liver paste increments, totaling five final mixtures. The nutritional compositions and physicochemical, microbiological, and toxicological characteristics of each mixture were assessed. The dry matter percentage of the prepared product was determined to be 27.00%, while mineral content was 1.45%, lipid content was 4.00%, and total protein content was 20.00%. Finally, microbiological counts were in agreement with current food safety regulations. The developed pâté serves as a standard, recycling underused industrial materials, adding value to the production chain at low operational costs, creating a more accessible market, and promoting the popularization of this type of meat.

## 1. Introduction

Pâté is an emulsified spreadable meat product consumed worldwide, especially in European countries, and widely accepted due to its rich flavor and smooth texture. This foodstuff is composed mainly of liver, lean meat, animal fat, and spices and is generally considered an added-value product with high nutritional and sensory qualities. The unique taste and texture of pâté can be attributed to the type and amount of incorporated fat, ranging between 17 and 50% [1,2,3,4,5].

Bullfrogs (*Aquarana catesbeiana*) are resistant to climatic variations, supporting even negative temperatures, and, although native to North America, are now found in over 41 countries worldwide. Commercial bullfrog production has increased or stabilized in recent years, with a 100% worldwide increase in 2018 compared to 2010. From 2010 to 2020, the world’s average bullfrog production was about 3200 tons per year, led by Taiwan, Malaysia, Singapore, Brazil, Ecuador, and Mexico [6]. 

Liver pâté is a traditional foodstuff manufactured using pig or calf liver, porcine back fat, and other characteristic ingredients, introduced in several countries for commercial purposes [7]. In this regard, frog meat contains high protein and adequate amino acid contents, as well as low lipid levels and high polyunsaturated fatty acid percentages, resulting in low fat and cholesterol products. The differentiated composition and nutritional value of this meat compared to other animal products attracts consumers and is beneficial in treating certain diseases and physiological disorders, mainly allergies [8,9].

Bullfrog carcasses consist of the thighs and torso (forechest). The thighs exhibit a high commercial value, while the torso, which accounts for nearly half of a bullfrog carcass’s weight, is cheaper due to a relatively high number of bones. Although the thighs represent the highest commercial bullfrog carcass value, their yield is lower than the other carcass portions, totaling about 40%. The front part is made up of breasts and arms and is usually neglected by consumers due to low meat yields. Bullfrog torsos exhibit the second-highest carcass yield (27%). However, although their use results in similar yields obtained as the front bullfrog carcass portions, torsos are not commercially relevant compared to thighs due to their bone structure. Several alternatives are, however, now being developed to better use the meat from these undervalued frog carcass portions associated with viscera. These include the development of novel products such as pâtés, sausages, and preserves. It is, however, important to emphasize the need to maintain adequate sensory characteristics in order to ensure consumer acceptance and accessibility [10,11,12].

The Mechanically Deboned Meat (Mechanically Separated Meat—MSM) technique is an alternative offered by the fishing industry to the industrial sector for product diversification, resulting in easy-to-digest foodstuffs rich in proteins and minerals, mainly including calcium and phosphorus and vitamins A, D, and complex B [12]. The upholding of the fishery industry in the current consumer market is directly associated with its ability to respond to new consumer demands, and the development of new types of fishery meat products with less impact on modern consumer routines has become a new requirement for healthy, differentiated, balanced, and easy to prepare foodstuffs with high nutritional value [1,10,13,14,15].

The development of new fishery-based products is favorable for the production of high nutritional value foodstuffs and should take advantage of the high amounts of rejected raw material to this end. In this sense, bullfrog meat consumption has become significantly relevant and is now the focus of research aiming at the development of new products, reinforcing food chain production [11,16]. In this regard, novel technologies have emerged to target and favor fishery products as food sources, transforming rejected meat into nutritious products with high market acceptance, such as surimi, pâtés, dumplings, nuggets, and fish burgers [1,10,13,14,15].

In this context, this study aimed to develop a pâté product based on a mixture of MSM from bullfrog (*Aquarana catesbeiana*) torso meat and liver paste mixed at different ratios and determine the nutritional composition and physicochemical, microbiological, and toxicological characteristics of the final mixtures.

## 2. Material and Methods

### 2.1. Liver Pâté Development

Bullfrog specimens were purchased from local farmers in Belo Horizonte, in the state of Minas Gerais, Brazil, between August and September 2019 and 2020. The bullfrogs were bought as chilled packs, stocked in isothermal containers, and taken to the Fish and Aquatic Health Laboratory at the Federal Fluminense University. The torsos and livers were homogenized employing an automatic meat grinder, resulting in the base minced meat used for all mixtures. Following the bleaching process, applied to facilitate bone removal, all meat portions were removed, ground, and homogenized. Torso meat and liver samples were assessed for quality characterization through physicochemical, microbiological, and toxicological analyses. A total of 250 g was taken from the processed raw material (base), the pre-tightening material (pâté paste), and the heat-treated products. All samples were packed, frozen, and separated in triplicate for each analysis. The processed liver, without the gallbladder and capsule, and the deboned bullfrog torso meat were both homogenized employing a cutting processor for 5 min. The pre-processed samples used to prepare the pâtés were packaged in vacuum food bags and frozen at −20 °C.

The torso and liver pâté products were prepared at the following ratios: 90% torso meat and 10% liver (Treatment 1—T1); 80% torso meat and 20% liver (Treatment 2—T2); 70% torso meat and 30% liver (Treatment 3—T3); 60% torso meat and 40% liver (Treatment 4—T4); and 50% torso meat and 50% liver (Treatment 5—T5). Finally, 2.0% sodium chloride was added to the T1 to T5 products, torso carcass meat (C1), and processed livers (C2). The five treatments were then subjected to heating at 121 °C for 30 min for product cooking and sterilization (15 min of cooking and 15 min of appertization). The final products were stored under refrigeration conditions. Each treatment was prepared in triplicate, with each batch containing 50 g of samples stored in individualized glass bottles.

The final samples were then placed in an isothermal container and transported to the State Center for Research and Food Quality (CEPQA) belonging to the Agricultural Research Company of the State of Rio de Janeiro (PESAGRO-RIO) and Laboratory of Physical–Chemical Control of Products of Animal Origin at the Federal Fluminense University for physicochemical, microbiological, and toxicological analyses.

### 2.2. Physicochemical Characterization

Physicochemical assays were carried out in triplicate following Brazilian Ministry of Agriculture, Livestock and Supply (MAPA) guidelines [17,18] based on the AOAC manual [19]. Hydrogen potential determinations were carried out using an ion phs-3e pHmeter (Satra^®^, Kettering, UK), and aqueous activity (Aw) was found through infrared analyses employing an Aqualab^®^ apparatus (São Paulo, Brazil) for bullfrog torso meat, liver, and the final mixtures. For the number of reactive substances to thiobarbituric acid (TBARs), the methodology used was described in the AOAC manual [19]. A 1 g sample was dissolved in 9 mL of HCl 0.25 meqL^−1^ solution containing 0.375 g 100 mL^−1^ of TBA (Sigma-Aldrich, St Louis, MO, USA). It was then mixed with the dilution of 15 g 100 mL^−1^ of tricloracetic (TCA) (Merck, Darmstadt, Germany), boiling the assembly for 10 min. The prepared was bulged with deionized water and centrifuged at 3500 rpm for 15 min (MIKRO20, Hettich Zentrifugan, Germany). The optical density of the supernatant was read at 532 nm using a UV/VIS Probe 1800 Shimadzu spectrophotometer (Shimadzu^®^, Kyoto, Japan), and the results expressed in mg of malonaldehyde (MA) kg^−1^ sample. Samples were first pre-dried at 105 °C to obtain water and total solid contents, and the pre-dried samples were then subjected to calcination to determine total mineral contents. Total crude protein contents were determined indirectly through a distillation method. Total lipid contents were obtained through an ether extract using a Goldfish extractor (Tecnal^®^, Piracicaba, Brazil). Individual minerals were determined using a GT-FC1IMP flame photometer (Global Equipamentos, Jaboticabal, Brazil), focusing on the most important minerals (K, P, Na, Mg, Ca, Zn, Fe, Cu, and Mn). Collagen contents were determined using a UV/VIS Probe 1800 Shimadzu spectrophotometer (Shimadzu^®^, Kyoto, Japan) employing hydroxyproline as the biological marker. 

### 2.3. Microbiological Characterization

Microbiological bullfrog torso meat and liver analyses were conducted to attest to the quality of the raw material after bleaching. Following the processing (day zero), the five treatments were subjected to microbiological analyses to attest to final product quality. Microbiological parameters were assessed in accordance with Brazilian guidelines for fish-based products [20] according to APHA methodologies [21]. 

Total coliforms, coliforms at 45 °C, coagulase-positive staphylococci, and *Salmonella* spp. were analyzed [18]. Total mesophilic bacteria counts were carried out to assess raw material and manufacturing quality. Serial dilutions were prepared at a 1:1000 ratio from 25 g of each sample. Each dilution was inoculated into Petri dishes containing specific culture media for each investigated microorganism. Viable cell counts after incubation were performed through plate counts using a Biocell Biocc150-Bi colony counter (Prolab^®^, São Paulo, Brazil). Microbiological analyses were repeated after 15 and 30 days to verify the quality of each mixture stored under refrigeration for shelf life assessments.

### 2.4. Toxicological Evaluation

The toxicological analyses comprised hydrogen sulfide (H_2_S) and histamine determinations ([19]). For histamine, 10 g of each sample was extracted using methanol and purified using Bio-Rad AG 1-X8 ion exchange columns (Bio-Ra^®^ Hercules, CA, USA). Histamine quantifications followed AOAC guidelines, employing a BK-UV1600 fluorescence spectrophotometer (Biobase^®^, Jinan, China) with excitation and emission wavelengths set at zx4 350 nm and 444 nm. Calibration curves were prepared using histamine solutions (Sigma-Aldrich, St. Louis, MO, USA) at 0.5 µg 5 mL^−1^, 1 µg 5 mL^−1^, 1.5 µg 5 mL^−1^, 2 µg 5 mL^−1^, and 2.5 µg 5 mL^−1^. Hydrogen sulfide detection was performed through a lead acetate reaction, where the presence of dark spots on filter paper indicates a positive result.

### 2.5. Statistical Analyses

Results are expressed as the means and standard error means (SEM) of three experiments encompassing triplicate determinations. All statistical analyses were performed using the Infostat version 2020 software (Infostat Software, Córdoba, Argentina) and were performed employing parametric parameters (mean, standard deviation, and maximum and minimum values) and tables. Statistical treatments were carried out by comparing means and frequencies employing the Tukey tests and frequency assessments (ANOVA) at a significance level of 5% (*p* ≤ 0.05) for treatment and experimental group comparisons [22].

## 3. Results and Discussion

### 3.1. Body Part Weight-Ratio and Carcass Utilization

Pâté yields were satisfactory, with approximately 100 g of carcass and liver required to produce 72 to 86 g of the different pâté ratios. On a larger scale, one ton of forechest and liver treated as subproducts could result in an average of 820 kg of a product presenting excellent performance and added value, minimizing the amounts discarded from processed carcasses. Compared with other products, 1.2 kg of raw *Piaractus brachypomus* meat allows for the production of only about 700 g of processed pâté [16]. Notably, the entire proposal presented herein employs an efficient use of animal parts with low commercial value, resulting in a final product with low associated costs.

The weight ratio of bullfrog carcasses was determined by relative forechest, thigh, calf, and trunk meat amounts, established as 12.70%, 41.12%, 20.27%, and 25.91%, respectively. The trunk is the second most abundant portion, although it contains relatively little meat compared to the thighs, calves, and forechest, which contain the main edible bullfrog meat portions. A 38.61% carcass yield corresponding to undervalued animal parts was noted, greatly reducing added product value due to underutilization. 

Different muscle regions and the liver usually have little or no commercial use and are usually discarded. Remodeling the classification of carcass products marketed for different purposes makes it more economical to use these routinely discarded portions. Similar carcass yields and ratios are noted compared to other studies that evaluate the use of animal cuts and their nutritional composition [11,12]. The academic literature consistently affirms the presence of a relationship between a favorable carcass yield and high-quality meat, be it in terms of nutritional parameters or meat quality [23]. Prioritizing the utilization of the majority of the animal’s anatomical parts enables the incorporation of sections that exhibit favorable meat quality.

### 3.2. Development and Characterization of Bullfrog Pâtés

The final products presented a characteristic bullfrog meat and liver odor and a dark coloration due to liver incorporation. Understanding the relationship between the composition and characterization of the final mixtures and technological functionality is essential to understanding how ingredient ratios and technological processing contribute to desirable physical–chemical and sensory characteristics [1,24]. 

It is important to highlight liver use during pâté processing following heat treatment. Liver proteins exhibit functional properties of significant technological interest, as they exhibit solubilization, gel foam, and emulsification capacity, an important final product characteristic. Liver proteins also exhibit antioxidant properties capable of preventing lipid oxidation, making the use of food additives, such as thickeners, in final products unnecessary [25].

These features are applied in improving novel products derived from meat co-products while also reducing shear strength and improving product texture. The bullfrog meat and liver mixture applied herein guarantees final food product quality and the enhancement of certain technical–functional characteristics, such as foaming capacity and stability. Studies assessing the physicochemical characteristics of pâté containing pork and poultry liver have noted that liver incorporation also aids in nutritional characteristic preservation, even when subjected to heat treatment, guaranteeing desirable product characteristics, such as high essential amino acid and unsaturated fat contents with functional characterization [26].

Liver incorporation is focused not only on the use of viscera but mainly on the addition of fat and protein to the final product. Future studies aimed at selecting formulas with desired characteristics will be developed based on our findings for the subsequent incorporation of new ingredients and sensory analyses.

### 3.3. Macro- and Micronutrient Pâté Contents 

No significant Aw and pH differences were observed during the processing period, with average Aw values of 0.972 ± 0.04 and pH values ranging from 7.0 to 6.6. Individual parameters are presented in Table 1 below. 

The decreased pH values observed in each treatment are directly associated with the reduced liver percentages added to each formulation. The final product pH is directly associated with the interaction between lipids and the aqueous product portion, as reduced liver amounts result in decreased lipid and aqueous portion interactions. The literature also describes that this interaction varies according to the type of liver during emulsification, where chicken liver exhibits the potential to increase pH product values [27]. Fat effects on meat and meat product textures have been extensively studied, with higher fat contents associated with less firm and more juicy products [28].

Food Aw and pH variables are directly associated with rancidity and protein degradation. Fishery meat products are considered a source of desirable fatty acids and valuable nutritional components, with the color deterioration caused by lipid oxidation comprising the main quality degradation factor during food processing and storage [2,29]. 

As mentioned previously, the literature has demonstrated that the association of proteins and lipids during pâté emulsification mainly prevents lipid oxidation, ensuring a greater supply of desired fatty acids for consumers and increasing product shelf lives [25,30].

The macronutrient composition values of the prepared products and the bullfrog torso meat and liver paste are displayed in Table 2.

Frog carcass heating (bleaching) at 60 °C for 15 min followed by deboning allowed for suitable raw meat structure, composition, and nutritional quality, confirmed by the water content, ash, protein, and lipids results. Homogenizing the final product base mass (mixed pulp) for pâté production is noteworthy, as no final product appearance changes were noted. No intrusive odor was noted in the final products, ensuring food quality. Furthermore, all samples were homogeneous and contained no solid residues, which could lead to handling or consumer risks [1,8]. 

Concerning fish pâté products, moisture, lipid, protein, ash, and carbohydrate contents of 61.2%, 22.2%, 11.6%, 2.2%, and 2.8%, respectively, have been reported for white croaker (*Micropogonias furnieri*) pâtés and contents of 61.4%, 19.2%, 10.6%, 4.5%, and 4.3%, respectively, for cachama (*Piaractus brachypomus*) pâtés [1].

No significant dry matter differences were observed when comparing C1 with the five pâté treatments. The literature indicates that liver pâté incorporation does not affect dry matter content due to final product emulsification, contributing toward nutrient preservation [31].

The developed products exhibited low fat and high protein content. However, only a general comparison to other studies was possible, as previous assessments focused on the meat from whole bullfrog legs instead of forechest meat [12]. In addition, fat contents were slightly higher than reported in other studies, which may be associated with the added liver and the fact that the final products comprised fixed liver and animal back segment ratios. The high water content of bullfrog meat makes this meat more tender and softer than other meats. In addition, bullfrog meat fat content is low, especially forechest meat, thus making it a good choice for people who require low fat but high protein intakes.

The total amino acid and collagen contents reported herein are also noteworthy, with total essential amino acids (EAAs) ranging from 8 to 9% in relation to total crude protein content in the mixed matrix and collagen ranging from 12 to 15%. Most meat properties were maintained, mainly concerning the essential amino acids lysine and methionine. The total amino acid contents of different bullfrog meat parts range from 10.30% to 15.41%, according to Zhu et al. [12]. Considering the different applied mixture ratios and heat processing, no significant protein loss was observed compared to torso meat and liver samples. Similar EAA ranges have been described in the literature, reinforcing the potential use of bullfrog livers and meat mixtures as high-quality protein sources.

Thigh meat is richer in essential amino acids when compared with bullfrog forechest and calf muscle meat. Amino acids are the most important taste compounds in food, and bullfrog meat is favored in this regard, with a unique flavor. Crude protein contents (17–24%), as well as the mono and dipeptide levels, were similar for the forechest, thighs, and calf (18.61%, 19.04%, and 18.98%, respectively). Zhu et al. [12] describe a wide distribution of functional monopeptides and essential amino acids in different muscle segments. The addition of liver to the developed pâté, however, allows not only for increased protein levels but also heightened functional product capacity. 

High protein foods of animal origin, especially developed from bullfrog carcasses, contain proteins with a high solubilization capacity due to high essential amino acid levels. The development of products from this raw material can also serve demanding consumers interested in foods with added technological value, such as products based on noble parts or by-products, which constitute promising alternatives. In this sense, pâté production is an established alternative in this regard, especially concerning low commercial value materials. Thus, technological development generates an alternative high-quality product developed from this by-product [12,16].

The main detected micronutrients were K, P, Na, Mg, Ca, Zn, Fe, Cu, and Mn (Table 3). Potassium was the most abundant mineral in all samples, followed by P, sodium, Mg, and Ca. Since most micronutrients are involved in biochemical reactions, the forechest meat contains lower K, P, and Mg contents compared to livers (*p* < 0.05), albeit non-significantly [11]. Minerals are an important part of metabolic reaction bioprocesses in living organisms. Potassium, for example, contributes to the intracellular ion balance, while sodium constitutes the main extracellular cation, assuming an important role in acid–base balances. Phosphorus, on the other hand, constitutes phospholipids and phosphoproteins [24].

Mineral elements within pâté are sourced from bullfrog bones and livers, which undergo a series of processing and heating procedures. After the treatment to achieve a paste-like consistency, enzymatic reactions are initiated but subsequently halted through heat treatment. This paste-like substance is incorporated into meat product formulations, allowing for the enrichment of biologically active calcium, potassium, magnesium, sodium, and phosphorus primarily [32]. Higher levels of potassium, magnesium, and phosphorus are observed mainly in the liver. However, the limited presence of potassium, copper, manganese, and zinc in the meat-bone paste leads to a reduction in the content of these minerals when the meat-bone paste is used as a substitute for beef liver in reformulated pâtés. Nevertheless, it is noteworthy that the increased addition of liver did not result in a proportional increase in the concentration of these elements in the final pâté product.

### 3.4. Microbiological and Toxicological Characterizations of the Formulated Pâté 

After plate counting using specific media for each microorganism studied, morphotinctorial and biochemical analyses were performed to characterize the colonies found. The microbiological parameters of the developed formulations are depicted in Table 4 below:

Regarding safety parameters, despite the risk of post-processing contamination, all products were considered safe according to current microbiological and toxicological standards. The choice of applying a quality standard for products aimed at human consumption to raw materials is due to the need to ensure final product safety, as raw material selection is an important factor in highly manipulated products [17,20]. 

Coliforms, enterococci, and staphylococci are common bacterial contaminants in fishery products, originating mainly in the handling stage. Thermotolerant coliforms, *Salmonella* spp., and coagulase-positive *Staphylococcus* present in the bullfrog raw material were all within Brazilian legislation standards from the handling stage to the storage period [8,10,20]. Adequate heat treatment contributes to significant microbial population reductions in final fish meat preparations [1].

The total mesophilic counts observed herein demonstrate the efficiency of the applied heat treatment. Total mesophilic counts above 2.95 log10CFU g^−1^ were detected in the raw material, undergoing a drastic reduction after thermal processing, present in minimal concentrations in the final products. Despite not being recommended in Brazilian legislation, total mesophilic bacteria counts comprise an important parameter to be evaluated during product development, are directly correlated with the entire processing quality, and may reflect on final product quality [33,34,35].

The microbiological evaluation results following the storage period are presented in Table 5.

No microbial growths were noted in any of the evaluated treatments after a 30-day storage period, indicating product suitability under adequate storage as a result of heat treatment effectiveness, which was shown as capable of completely inactivating initial viable cells associated with adequate storage, prolonging their shelf life. Heating and chilling processes are applied to food preservation with the aim of inactivating and preventing microbial growth, serving as a basis for shelf life assessments [36,37,38]. They may also, however, result in product quality risks, as they may alter sensorial and nutritional product characteristics [39].

The toxicological paremeters evaluated are presented in Table 6 below:

No histamine and sulfur-based compounds were detected in any of the samples, with an established limit of detection of 1.00 µg g^−1^, in accordance with the Brazilian standard of 10 mg 100 g^−1^ [17] and the North American standard of 5 mg 100 g^−1^ [40]. A value of 1 mg 100 g^−1^ histamine has been previously reported for thermally processed bullfrog meat [9]. Therefore, both the raw bullfrog materials and the final products formulated herein presented satisfactory hygienic-sanitary conditions, with no significant handling and processing interferences concerning product safety.

## 4. Conclusions

The present study aimed to develop high nutritional potential foods using by-products that would normally be discarded by the industry through technologies commonly used during food processing, resulting in standardized and practical food products without losing functional and nutritional characteristics. The final bullfrog products, despite being prepared from highly perishable raw material and undergoing numerous manipulations, displayed adequate microbiological counts, nutritional parameters, and protein and lipid values, demonstrating the importance of applying good manufacturing practices when preparing fishery-based products to maintain product quality and avoid contamination, adding value to the production chain by recycling underused industrial materials. The results of product characterization analyses will serve as new standards for the development of novel products using fishery-based by-products, guaranteeing not only product nutritional quality but also safety. The valuation of other bullfrog carcass parts allows for the development of new foods using fewer noble parts with low operational costs and, consequently, a reduction in product prices, creating a more accessible market and promoting the popularization of this type of meat.

## Figures and Tables

**Table 1:** Hydrogen potential (pH), thiobarbituric acid reactive species (TBARS), and water activity (Aw) of minced bullfrog torso meat (*Aquarana catesbeiana*), liver paste, bullfrog meat, and liver pâtés prepared at different ratios.

Samples (*)	pH	TBARS (mg MDA kg^−1^)	Aw
Torso meat (C1)	6.8 ± 0.10 ^A^	1.60 ± 1.10 ^A^	0.970 ± 0.10 ^A^
Liver paste (C2)	6.60 ± 0.10 ^A^	1.70 ± 1.10 ^A^	0.980 ± 0.10 ^A^
Mixed pulp (T1)	6.82 ± 0.10 ^A^	1.40 ± 1.10 ^A^	0.960 ± 0.10 ^A^
Mixed pulp (T2)	6.72 ± 0.10 ^A^	1.30 ± 1.10 ^A^	0.960 ± 0.10 ^A^
Mixed pulp (T3)	6.78 ± 0.10 ^A^	1.20 ± 1.10 ^A^	0.970 ± 0.10 ^A^
Mixed pulp (T4)	6.99 ± 0.10 ^A^	1.20 ± 1.10 ^A^	0.960 ± 0.10 ^A^
Mixed pulp (T5)	7.00 ± 0.10 ^A^	1.20 ± 1.10 ^A^	0.960 ± 0.10 ^A^

(*) Means (n = 3) of triplicate analyses. Control (C1 and C2); 90% torso meat and 10% liver (T1); 80% torso meat and 20% liver (T2); 70% torso meat and 30% liver (T3); 60% torso meat 40% liver (T4); and 50% torso meat and 50% liver (T5). A represent significant differences according to the Tukey test (*p* ≤ 0.05).

**Table 2:** Proximate composition of minced bullfrog torso meat (*Aquarana catesbeiana*), liver paste, and bullfrog meat and liver pâtés prepared at different ratios.

Samples (*)	Dry Matter (%)	Crude Protein (%)	Collagen (%)	Lipids (%)	Ash (%)
Torso meat (C1)	27.20 ± 1.30 ^B^	25.60 ±1.04 ^B^	13.22 ± 1.04 ^B^	4.10 ± 0.31 ^B^	1.55 ± 0.14 ^A^
Liver paste (C2)	12.90 ± 1.10 ^A^	17.80 ± 2.16 ^A^	0.10 ± 0.01 ^A^	6.20 ± 0.81 ^A^	1.15 ± 0.25 ^A^
Mixed pulp (T1)	27.42 ± 1.46 ^B^	24.50 ± 1.10 ^B^	11.25 ± 1.10 ^B^	4.50 ± 0.62 ^B^	2.22 ± 0.25 ^B^
Mixed pulp (T2)	28.72 ± 0.96 ^B^	23.33 ± 1.61 ^B^	12.53 ± 1.54 ^B^	4.44 ± 1.84 ^B^	2.08 ± 0.39 ^B^
Mixed pulp (T3)	28.78 ± 2.10 ^B^	21.27 ± 0.91 ^B^	11.50 ± 1.22 ^B^	3.66 ± 0.71 ^B^	2.54 ± 0.16 ^B^
Mixed pulp (T4)	27.99 ± 0.59 ^B^	19.14 ± 1.29 ^B^	12.20 ± 1.26 ^B^	3.79 ± 1.25 ^B^	2.14 ± 0.35 ^B^
Mixed pulp (T5)	26.97 ± 1.00 ^B^	19.10 ± 0.88 ^B^	12.10 ± 1.23 ^B^	4.67 ± 0.79 ^B^	1.99 ± 0.30 ^B^

(*) Means (n = 3) of triplicate analyses. Control (C1 and C2); 90% torso meat and 10% liver (T1); 80% torso meat and 20% liver (T2); 70% torso meat and 30% liver (T3); 60% torso meat 40% liver (T4); and 50% torso meat and 50% liver (T5). A and B represent significant differences in each column according to the Tukey test (*p* ≤ 0.05).

**Table 3:** Potassium (K), calcium (Ca), phosphorus (P), sodium (Na), magnesium (Mg), and copper (Cu) of minced bullfrog torso meat (*Aquarana catesbeiana*), liver paste, bullfrog meat, and liver pâtés prepared at different ratios.

Samples (*)	K (%)	Ca (%)	P (%)	Na (%)	Mg (%)	Cu (%)
Torso meat (C1)	0.200 ± 0.11	0.550 ± 0.14	0.005 ± 0.009	0.001 ± 0.001	0.001 ± 0.001	0.001 ± 0.001
Liver paste (C2)	0.500 ± 0.12	0.050 ± 0.01	0.200 ± 0.110	0.001 ± 0.001	0.001 ± 0.001	0.001 ± 0.001
Mixed pulp (T1)	0.22 ± 0.12	0.22 ± 0.20	0.015 ± 0.010	0.051 ± 0.010	0.001 ± 0.001	0.001 ± 0.001
Mixed pulp (T2)	0.27 ± 0.12	0.18 ± 0.12	0.016 ± 0.010	0.052 ± 0.010	0.001 ± 0.001	0.001 ± 0.001
Mixed pulp (T3)	0.34 ± 0.16	0.34 ± 0.16	0.013 ± 0.010	0.051 ± 0.010	0.001 ± 0.001	0.001 ± 0.001
Mixed pulp (T4)	0.34 ± 0.10	0.24 ± 0.20	0.012 ± 0.010	0.051 ± 0.010	0.001 ± 0.001	0.001 ± 0.001
Mixed pulp (T5)	0.29 ± 0.10	0.29 ± 0.20	0.017 ± 0.010	0.053 ± 0.010	0.001 ± 0.001	0.001 ± 0.001

(*) Means (n = 3) of triplicate analyses. Control (C1 and C2); 90% torso meat and 10% liver (T1); 80% torso meat and 20% liver (T2); 70% torso meat and 30% liver (T3); 60% torso meat 40% liver (T4); and 50% torso meat and 50% liver (T5).

**Table 4:** Microbiological characterization of the different bullfrog pâté formulations developed herein.

Microorganisms	Brazilian Legislation *	C1	C2	T1	T2	T3	T4	T5
Total mesophilic bacteria ^1^	-	5.50	2.95	<1.00	<1.00	<1.00	<1.00	<1.00
coagulase-positive *Staphylococcus* ^1^	3.00	2.60	3.00	<1.00	<1.00	<1.00	<1.00	<1.00
Total coliforms ^1^	-	2.50	2.50	<1.00	<1.00	<1.00	<1.00	<1.00
Thermotolerant coliforms ^1^	2.70	1.55	1.55	<1.00	<1.00	<1.00	<1.00	<1.00
*Salmonella* spp. ^1^	Abs.	Abs.	Abs.	Abs.	Abs.	Abs.	Abs.	Abs.

*: Microbiological standards according to Brazilian legislation [20]; ^1^: CFU counts expressed in log_10_CFU g^−1^; Limit of detection (LOD) = < 1.0 log_10_CFU g^−1^; Abs. = absent.

**Table 5:** Microbiological evaluation results following chilled storage.

Microorganisms	Brazilian Legislation *	15 Days of Storage					30 Days of Storage				
		T1	T2	T3	T4	T5	T1	T2	T3	T4	T5
Total mesophilic bacteria ^1^	-	<1.00	<1.00	<1.00	<1.00	<1.00	<1.00	<1.00	<1.00	<1.00	<1.00
coagulase-positive *Staphylococcus* ^1^	3.00	<1.00	<1.00	1.00	<1.00	<1.00	<1.00	<1.00	<1.00	<1.00	<1.00
Total coliforms ^1^	-	<1.00	<1.00	<1.00	<1.00	<1.00	<1.00	<1.00	<1.00	<1.00	<1.00
Thermotolerant coliforms ^1^	2.70	<1.00	<1.00	<1.00	<1.00	<1.00	<1.00	<1.00	<1.00	<1.00	<1.00
*Salmonella* spp. ^1^	Absent	Abs.	Abs.	Abs.	Abs.	Abs.	Abs.	Abs.	Abs.	Abs.	Abs.

*: Microbiological standards according to Brazilian legislation (BRASIL, 2022); ^1^: CFU counts expressed in log_10_CFU g^−1^; Limit of detection (LOD) = < 1.0 log_10_CFU g^−1^; Abs. = absent.

**Table 6:** Histamine levels and Sulfur-based (H_2_S) compounds of minced bullfrog torso meat (*Aquarana catesbeiana*), liver paste, bullfrog meat, and liver pâtés prepared at different ratios.

Samples (*)	Histamine (µg g^−1^)	H_2_S (mg 100 g^−1^)
Torso meat (C1)	≤1.00	≤1.00
Liver paste (C2)	≤1.00	≤1.00
Mixed pulp (T1)	≤1.00	≤1.00
Mixed pulp (T2)	≤1.00	≤1.00
Mixed pulp (T3)	≤1.00	≤1.00
Mixed pulp (T4)	≤1.00	≤1.00
Mixed pulp (T5)	≤1.00	≤1.00

(*) Means (n = 3) of triplicate analyses. Control (C1 and C2); 90% torso meat and 10% liver (T1); 80% torso meat and 20% liver (T2); 70% torso meat and 30% liver (T3); 60% torso meat 40% liver (T4); and 50% torso meat and 50% liver (T5).

## Data Availability

The data used to support the findings of this study can be made available by the corresponding author upon request.
